# Vortex fluidic mediated food processing

**DOI:** 10.1371/journal.pone.0216816

**Published:** 2019-05-30

**Authors:** Shan He, Nikita Joseph, Xuan Luo, Colin L. Raston

**Affiliations:** 1 Department of Food Science, School of Chemistry and Chemical Engineering, Guangzhou University, Guangzhou, Guangdong, China; 2 Flinders Institute for NanoScale Science and Technology, College of Science and Engineering, Flinders University, Bedford Park, South Australia, Australia; University of New South Wales, AUSTRALIA

## Abstract

The high heat and mass transfer, and controlled mechanoenergy, in angled vortex fluidics has been applied in chemical and material sciences and allied fields, but its utility in food processing remains largely unexplored. Herein we report three models of food processing incorporating such vortex fluidics, including enzymatic hydrolysis, raw milk pasteurization and encapsulation. The processing times of enzymatic hydrolysis was reduced from about 2–3 hours to 20 minutes, with the processing time of raw milk pasteurization reduced from 30 to 10 minutes, and an encapsulated particle size reduced approximately 10-fold, from micro meters to hundreds of nanometers. These findings highlight exciting possibilities, in exploiting the value of vortex fluidic mediated processing in the food industry.

## 2.Introduction

The advance of modern technologies has significantly improved the traditional methods of food processing. This has resulted in shorter processing times as well as the development of “new food” with better nutritional or functional values than traditional food products. For example, protein hydrolysates with smaller molecular weights from fish or milk [1,2] enhance its absorbance in the body, therefore increasing its nutritional values [1] and yoghurt, cheese or other traditional food with encapsulated fish oil or other nutritional substances is possible without destabilizing the food system. In comparison with the traditional pasteurization method, the application of advanced Ultra Heat Temperature (UHT) pasteurization has significantly increased the shelf life of milk without reducing its nutritional value [3].

Nevertheless, there are a number of challenges in the current food processing industries. Most of these relate to the balance between the quality of the final products and the cost of production. While protein hydrolysis from fish, milk and other protein sources provided the protein-based food with higher nutritional value, the processing time required to hydrolyze food protein into small peptides is about 2–3 hours [4]. This is at odds with food industry practice and cost considerations, requiring an acceptable processing time within 0.5 hours [5]. Regarding encapsulation of small particles, this results in instantaneous release of flavor and functioning of the confined substances. Currently it is difficult for conventional homogenization to generate particle-sizes at the nano-meter dimensions, unless operating the processing at high pressures, as reported by [6] for generating nano-crystalline suspensions under such conditions, with the particles approximately 200 nm in diameter. However, the high cost of a high pressure homogenizer (approximately USD 150k) limits its application mainly to laboratory operations, rather than extending them into industry. Indeed, in comparison with the traditional pasteurization method, the application of advanced Ultra High Temperature (UHT) pasteurization has significantly increased the shelf life of milk without reducing its nutritional value. Nonetheless, an UHT system costs over USD 1 million and is therefore difficult for small farms and small businesses to implement, in comparison with the cost of only USD 15,000 for establishing a traditional pasteurization system [2]. Therefore, an innovative low cost system with dramatically improved heat transfer and/or high shear has the potential to revolutionize the food processing industry [7].

**Fig 1:**
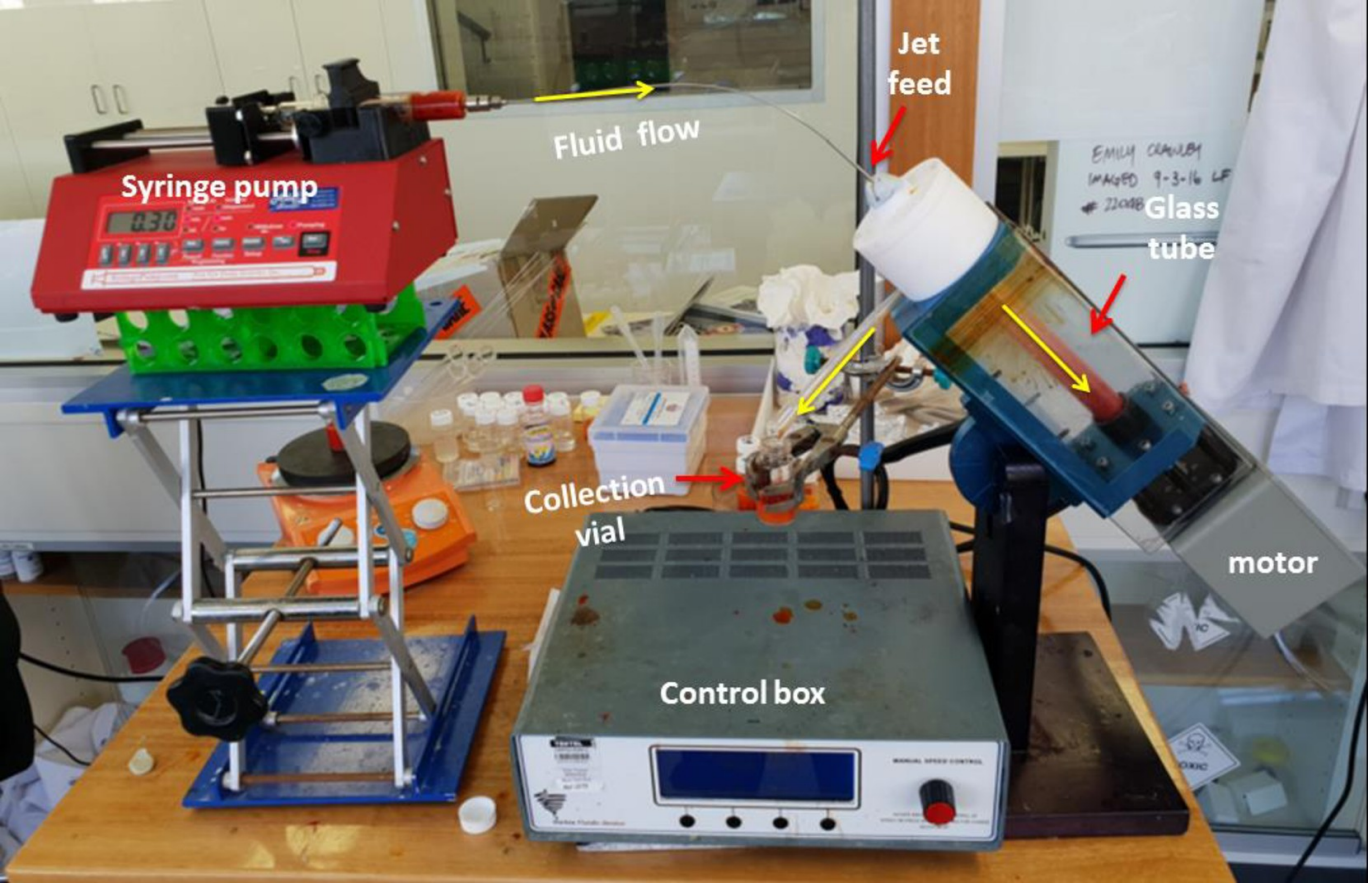
Photograph of the vortex fluidic device (VFD) highlighting its salient features.

Vortex fluidics represents a new processing capability that enables new tools and synthetic strategies with a diversity of research and industrial applications. Core processing technology has emerged from research efforts focused on the application of thin film microfluidics and thin film flow chemistry. This has resulted in the ability to harness high shear forces, intense micro mixing, and high heat transfer to enhance and explore reactivity relative to traditional batch processing [8]. This led to the development of the Vortex Fluidic Device (VFD) (Fig 1) which has a rapidly growing number of processing capabilities, from small molecule synthesis to processing advanced materials, for various applications such as in drug delivery, and manipulating single cell organisms [9]. The VFD can operate under the confined mode of operation where a tube containing a finite volume of liquid is spun at high speed, or operate under continuous flow where jet feeds deliver liquid to the base of the tube or at positions along the tube under the same conditions. A dynamic thin film is formed at high rotational speeds with the thickness of the thin film controlled by varying the rotational speed and tilt angle of the tube or the volume of liquid in the VFD tube, which under continuous flow, depends on the flow rate [8].

The utility of the VFD has been established for a number of chemical transformations, with control over reactivity and selectivity, and the ability to prepare complex molecules in a single pass in the thin film microfluidic platform. Dramatically enhancing enzymatic reactions has been established, with an average seven-fold acceleration for a diversity of enzymes [10]. This arises from the Faraday pressure waves contained within the thin film. Tethering enzymes to the surface of the tube has also been established, for the synthesis of small molecules in a single pass [9]. The VFD has also been applied in materials processing under continuous flow, such as in assembling fullerene C_60_ molecules into nanotubules [11]. The micron length nanotubules, with hollow diameters 100 to 400 nm, are formed in the absence of surfactant, and without the need for further downstream processing. The operating parameters of VFD can be systematically explored, as high throughput processing, in optimizing any process, as in effectively converting sunflower oil to biodiesel at room temperature, with no saponification and avoiding the conventional use of co-solvent or complex catalysts [12]. The thin film generated by the VFD significantly increases heat transfer and shear forces, which are two critical conditions/requirements in food processing, as discussed above. Thus far, the VFD as the showcase tool for vortex fluidics, has not been applied in food processing, other than preliminary findings on the encapsulation of fish oil [13]. This involved operating the VFD under continuous flow for controlling the size of spheroidal encapsulated fish oil particles, from 50 to 250 nm. Moreover, use of homogenization (as a benchmark to encapsulate fish oil) afforded much larger macro-particles, ranging in size from 2 to 4 μm. Smaller encapsulated particles fabricated using VFD processing could lead to improved absorption from fish oil.

Herein we explore the utility of the VFD thin film microfluidic platform in food processing, targeting three models of food processing, namely enzymatic hydrolysis, pasteurization and encapsulation. The effects of VFD processing are compared with the traditional processing technology. Overall, the data in this communication letter establishes a conceptual basis with respect to the application of the VFD in food processing.

## 3.Materials and methods

### 3.1 Materials

Both milk powder (Coles, Australia) and fish oil (Swiss, Australia) were food grade and purchased from local markets. Neutrase was purchase from Novozyme Australia (U3/22 Loyalty Road, North Rocks, NSW, 2151). Raw milk (unpasteurised) was provided by Bd farm Paris Creek PTY LTD (9/7 Paris Creek Rd, Paris Creek SA 5201) 24 hours before the commencement of the trial processing. Sucrose monolaurate and curcumin were purchased from Sigma-Aldrich, Australia (12 Anella Avenue, Castle Hill, NSW, 2154)

### 3.2 Methods

#### 3.2.1 Enzymatic hydrolysis of protein in milk powder

Ten grams of milk powder was fully dissolved in 50 mL of water. One percent Neutrase (w/w, enzyme to milk powder, 0.1 g) was added in the milk powder solution. For the conventional enzymatic hydrolysis method, 1 mL of the dissolved milk powder along with the enzyme was transferred into a 5 mL container and heated at 55°C for 2 hours using a water bath, followed by heating at 95°C for 15 minutes to deactivate the enzyme. For VFD enzymatic hydrolysis method, 1 mL of the dissolved milk with enzyme was transferred into the VFD tube (20 mm O.D. and 17.5 mm I.D., 18.5 cm in length). The confined mode of operation of the VFD was used, with the tube spun at 8000 rpm for 20 minutes, at a tilt angle of 45 degrees. The processing conditions of 8000 rpm rotation speed and tilt angle of 45 degrees were recognized as the optimal processing conditions for a number of previous VFD studies [14,9]. The temperature of the VFD tube was maintained at 55°C using a heating jacket. After that, the temperature of the VFD tube was elevated to 95°C and maintained at this temperature for 15 minutes in the VFD to deactivate the enzymes. Matrix-assisted laser desorption/ionization (MALDI) mass spectrometry was conducted for both the liquid samples after hydrolysis of the dissolved raw milk powder, for determining the molecular weights, which were used as the indicator of hydrolysis efficiency.

#### 3.2.2 Curcumin encapsulation with fish oil and sucrose monolaurate as an emulsifier

Nano-encapsulations were formulated using curcumin, as a bioactive ingredient, and a mixture of non-ionic surfactant and water. Briefly, 10 mg of curcumin was mixed and dissolved in 60 mg of fish oil. Then the curcumin-fish oil mixture was premixed with 10 mg of sucrose monolaurate and 10 mL of water by using a benchtop vortex mixer, and introduced into the borosilicate glass tube (20 mm OD) in the VFD through jet-feeds, with the tube rotating at 8000 rpm, at a flow-rate of 0.1 mL/min, with the tilt angle of the tube at 45° and the device operating at room temperature. The solution was collected and sonicated for 20 minutes. The traditional homogenization method was also applied for comparison with the VFD processing. Here the same concentrations as used above in the VFD were homogenized (homogenizer T25 digital ULTRA-TURRAX) at 13,500 rpm for 10 minutes at 25°C and similarly the homogenized solution was sonicated for 20 minutes.

#### 3.2.3 Particle size measurements

Oil in water (o/w) emulsions particle size distribution and polydispersity index were determined at 25°C using dynamic light scattering (DLS) (Nano ZS90, Malvern instruments, Worcester, UK) operating with a He-Ne 633 nm wavelength laser and a detector angle of 173°. Three independent measurements were performed for each sample. The Malvern zeta sizer instrument measured the time dependent fluctuations of light scattered based on the particle sizes. All the o/w emulsions were diluted in 1:10 with Milli Q water for all measurements.

#### 3.2.4 UV-visible absorption spectroscopy measurements

The absorption spectra for all the samples were measured on Cary 50 Bio UV-visible spectrophotometer at 25°C. The maximum absorption of curcumin was recorded at pH 7 at 25°C using a quartz cuvette of path-length 10 mm.

#### 3.2.5 Fluorescence spectroscopy measurements

All the fluorescence measurements were carried out with a Cary Eclipse Fluorescent Spectrophotometer, Agilent Technologies, using a quartz cuvette with a path-length 10 mm. Both excitation and emission band slits were fixed at 10 nm and the scan rate was selected at 1800 nm/min. The excitation wavelength was selected at 425 nm while emission spectra were collected in the range of 450 to 600 nm.

#### 3.2.6 Raw milk pasteurization

For the conventional processing, 1 mL of raw milk was sealed in a 5 mL container and pasteurized at 60°C for 30 minutes in a water bath. VFD processing used the confined mode with 1 mL of raw milk transferred into a VFD tube and spun at 8000 rpm with the tilt angle at 45°. For different trials, the heating temperatures were set at 60°C, 50°C and 40°C, and for each temperature the VFD was operated for 30, 20, 10 and 5 minutes, respectively. Alkaline Phosphatase tests were carried out on all pasteurized samples for determining the efficiency of the pasteurization.

#### 3.2.7 Measurement tests

MALDI mass spectrometry was used to determine the enzymatic hydrolysis efficiency, and Alkaline Phosphatase tests were used to determine the pasteurization efficiency, carried out according to standard methods.

## 4. Results and discussions

### 4.1 Enzymatic hydrolysis of protein in milk powder

**Fig 2:**
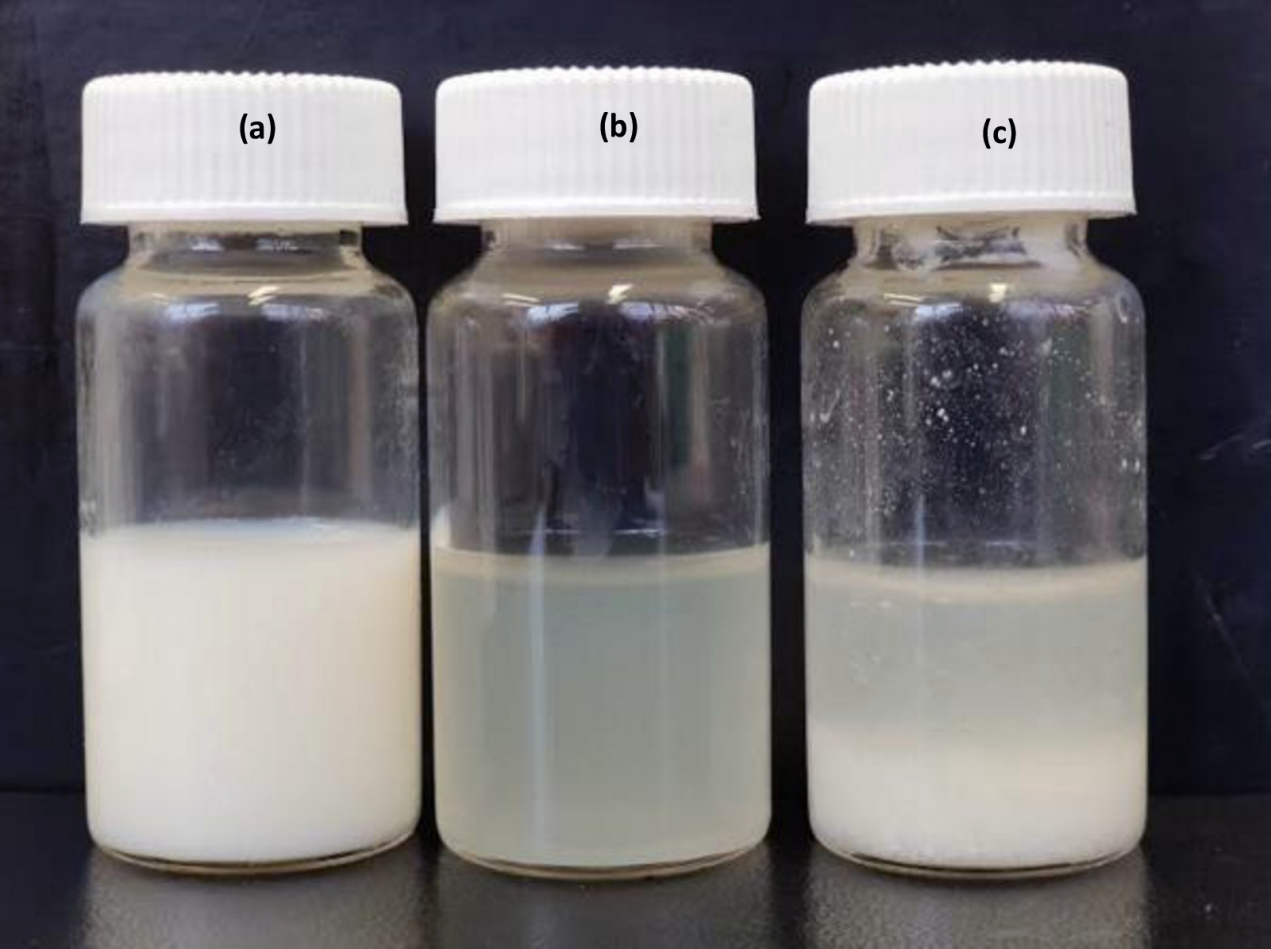
Appearance of dissolved milk powder with **(a)** no treatment; milk powder: water = 1:5 (w/v). **(b)** VFD-enzymatic hydrolyzation; milk powder: water = 1:5 (w/v); temperature at 55°C; processing time of 20 mins; VFD speed at 8000 rpm; VFD tilt angle of 45°. **(c)** Conventional enzymatic hydrolyzation; milk powder: water = 1:5 (w/v); temperature at 55°C; processing time of 20 min.

**Fig 3:**
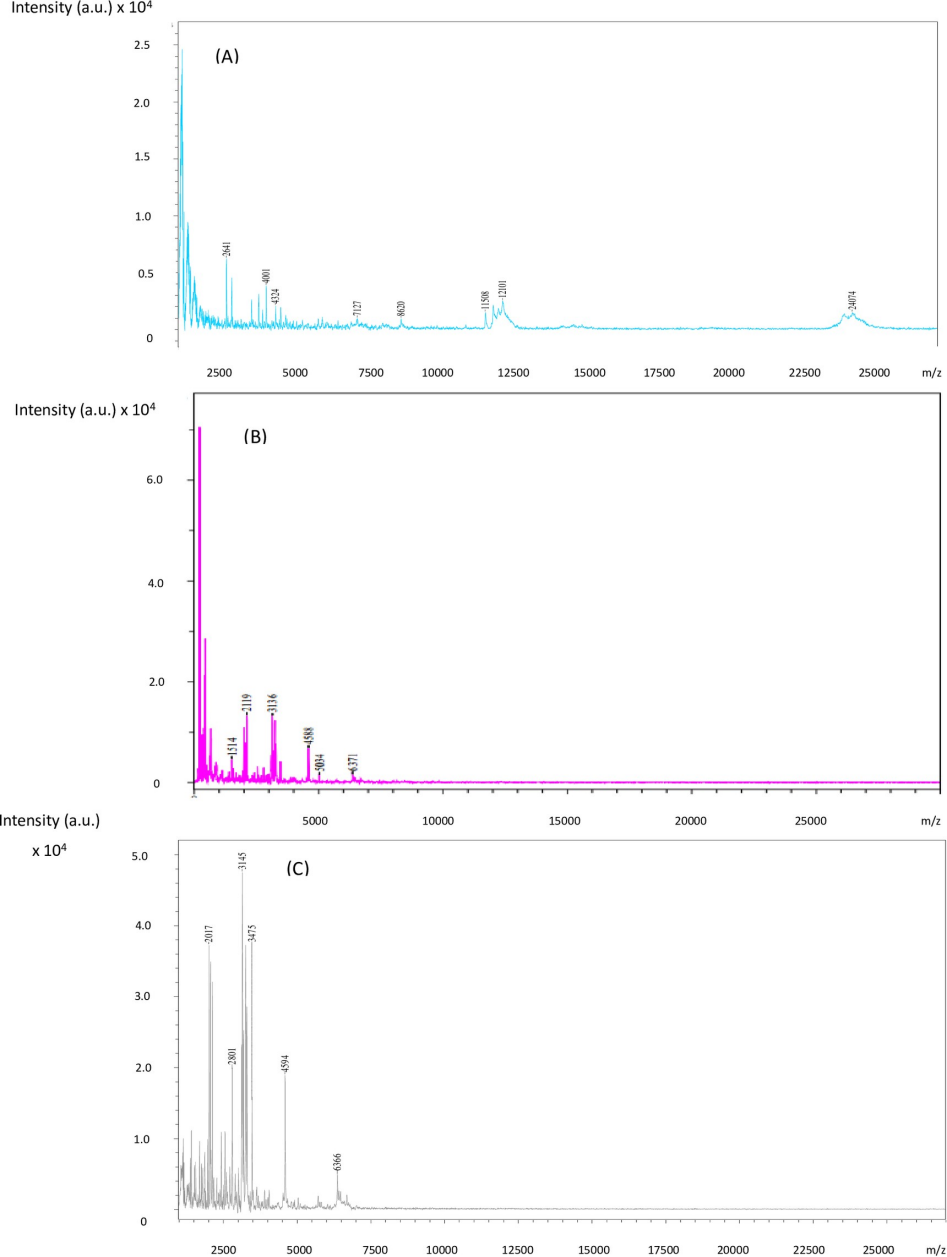
MALDI mass spectra analysis of dissolved milk powder before and after different treatments **(a)** No treatment; milk powder: water = 1:5 (w/v). **(b)** VFD-enzymatic hydrolyzation; milk powder: water = 1:5 (w/v); temperature at 55°C; processing time of 20 mins; VFD speed at 8000 rpm; VFD tilt angle at 45°. **(c)** Conventional enzymatic hydrolyzation; milk powder: water = 1:5 (w/v); temperature at 55°C; processing time of 20 min.

The appearances of dissolved milk powder, dissolved milk powder after VFD-enzymatic hydrolysis, and dissolved milk powder after conventional enzymatic hydrolysis are shown in Fig 2. The hydrolyzed protein after enzymatic hydrolysis is a protein that has been partially broken down, and therefore possesses lower molecular weight in comparison with intact protein. The cloudiness of the liquid in Fig 2C might indicate poor efficiency of hydrolysis, as reported by [15]. In comparison, the clear solution of Fig 2B is consistent with improved efficiency of hydrolysis, even though the processing was much shorter (20 minutes compared with 2 hours for conventional processing). This observation was confirmed using MALDI mass spectrometry analysis. The peaks on the left and right side in Fig 3 indicate protein with the lowest and highest MW, respectively. Milk protein is composed of a mixture of proteins with various sizes with wide m/z values from 0 to 25000 (Fig 3A). Prior to processing, there were several peaks from 12500 to 25000 m/z for the non-processed dissolved milk powder (Fig 3A). However, these peaks were not observed after hydrolysis using either the VFD or conventional processing (Fig 3B and 3C). Comparing the two processing techniques, it can be seen that the peaks in Fig3B were more concentrated on the left than those in Fig 3C. This indicates that the MW of the dissolved milk powder after VFD-enzymatic hydrolysis is smaller than the dissolved milk after conventional enzymatic hydrolysis, thereby demonstrating greater efficiency of hydrolysis assisted by VFD processing. Enzymatic hydrolysis has been carried out in an industrial scale, although there are some concerns, including the cost of enzymes as reported by [5], and processing time. The current enzyme cost for the cellulose to ethanol process is approximately USD 5 per gallon ethanol. However, the market-acceptable price is USD 1.07 per gallon, which requires a 10 fold reduction in the cost of the enzyme [16]. As to processing time, this can be up to 3 hrs with the food industry preferring enzymatic hydrolysis processing time down to 20 min [5]. The current trend is to develop new technologies to increase the efficiency of enzymes, to reduce the amount of enzyme required and/or the processing time. [17] immobilized glucose oxidase on TiO_2_ /Polyurethane for removal of a dye, with the resulting decolourization efficiency increased from approximate 60% for no immobilization to almost 100%. [5] developed a microwave-assisted enzymatic process which increased the production yield from 42% to 62.5%, and shortened the processing time from 2hrs to 20 min. The development of VFD technologies in this study presented another technology with the potential to enrich this trend.

### 4.2 Curcumin encapsulation in O/W emulsions

**Fig 4:**
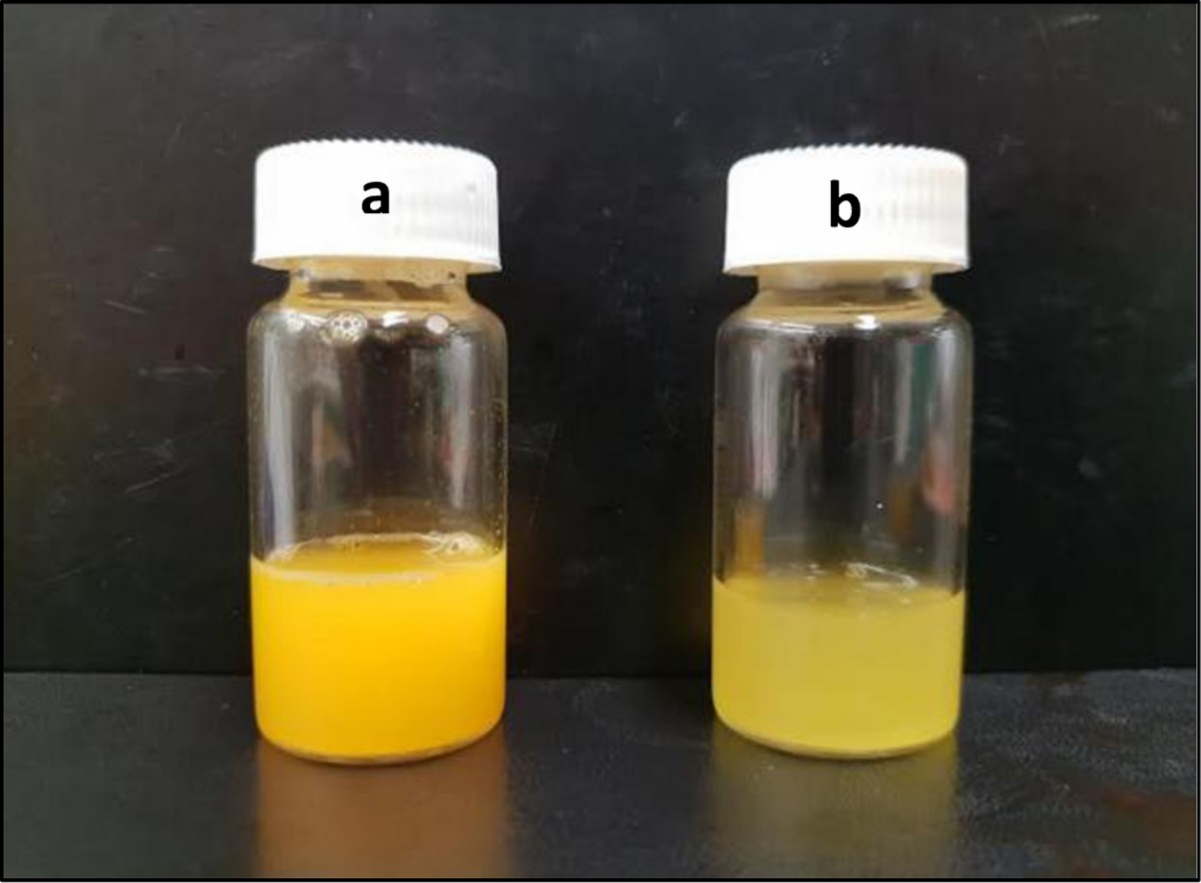
Photographs of different samples of encapsulated curcumin **(a)** Curcumin formulation processed using the homogenization method (speed: 13,500 rpm; time: 10 minutes; temperature: 25°C; sonication: 20 minutes; homogenizer: T25 digital ULTRA-TURRAX)**. (b)** Curcumin formulation processed using a vortex fluidic device (VFD) (dispersed phase: 2%(w/v), lipid/oil: 1/1(w/w), speed: 8000 rpm, flow-rate: 0.1 mL/min, tilt angle: 45°, temperature: 25°C; sonication post VFD processing for 20 minutes).

**Fig 5:**
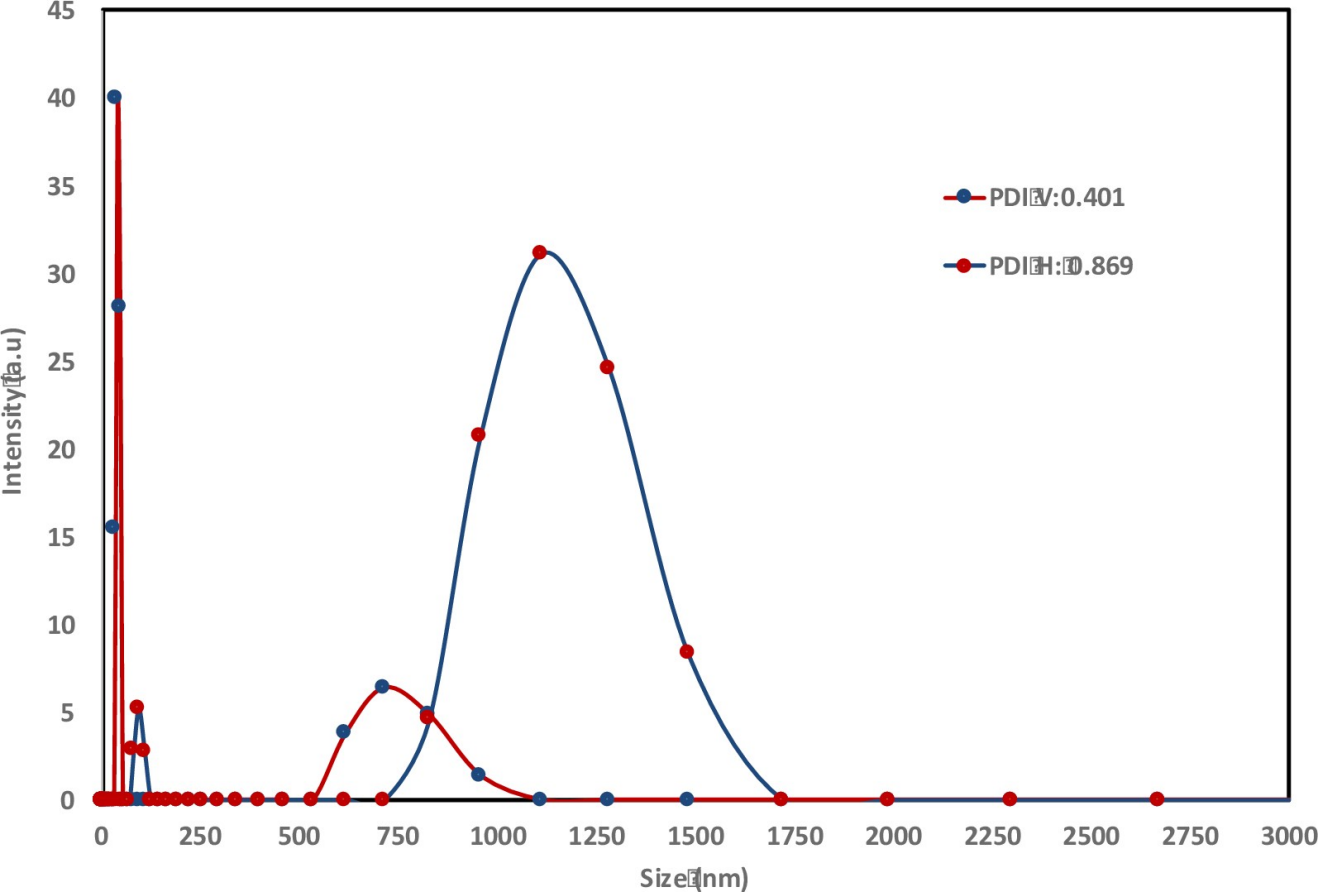
Dynamic light scattering (DLS) data (temperature: 25°C; He-Ne laser: 633 nm; detector angle: 173°) for encapsulated particles produced using vortex fluidic device (VFD) processing, using operating parameters in Fig 4 caption.

Fig 4 demonstrates that there are significant differences of particle size between the curcumin encapsulated by conventional homogenization method and VFD processing. Though there are 2 peaks shown for the diagrams of both conventional homogenization method and VFD processing, the conventional homogenization method produced capsules with the major size distribution above 1000 nm. In contrast, VFD processing significantly reduced the majority of the particles to the size of less than 100 nm. This finding is supported by the different appearance of curcumin encapsulated from different treatments. The solution of encapsulated curcumin processed using VFD processing was clearer (Fig 5A), whereas the solution of encapsulated curcumin processed by homogenization method was less transparent (Fig 5B). In this context, the particle size in solution is one of the decisive factors for transparency of solution or otherwise. [18] reported that the cloudiness of solution is drastically reduced once the particle size is less than 200 nm. A similar particle size of encapsulated curcumin has been previously reported [19]. However, to achieve this, high-pressure homogenization using a system costing about USD 150,000 was required. In contrast, the current cost of approximate USD 20,000 for VFD makes it a dramatically less expensive technology, and thus VFD processing represents a significantly cost saving processing, in achieving quality encapsulation. Scaling up of the VFD processing will be a focus of future studies, with the aim of improving the current industrial production of homogenization at large scale.

**Fig 6:**
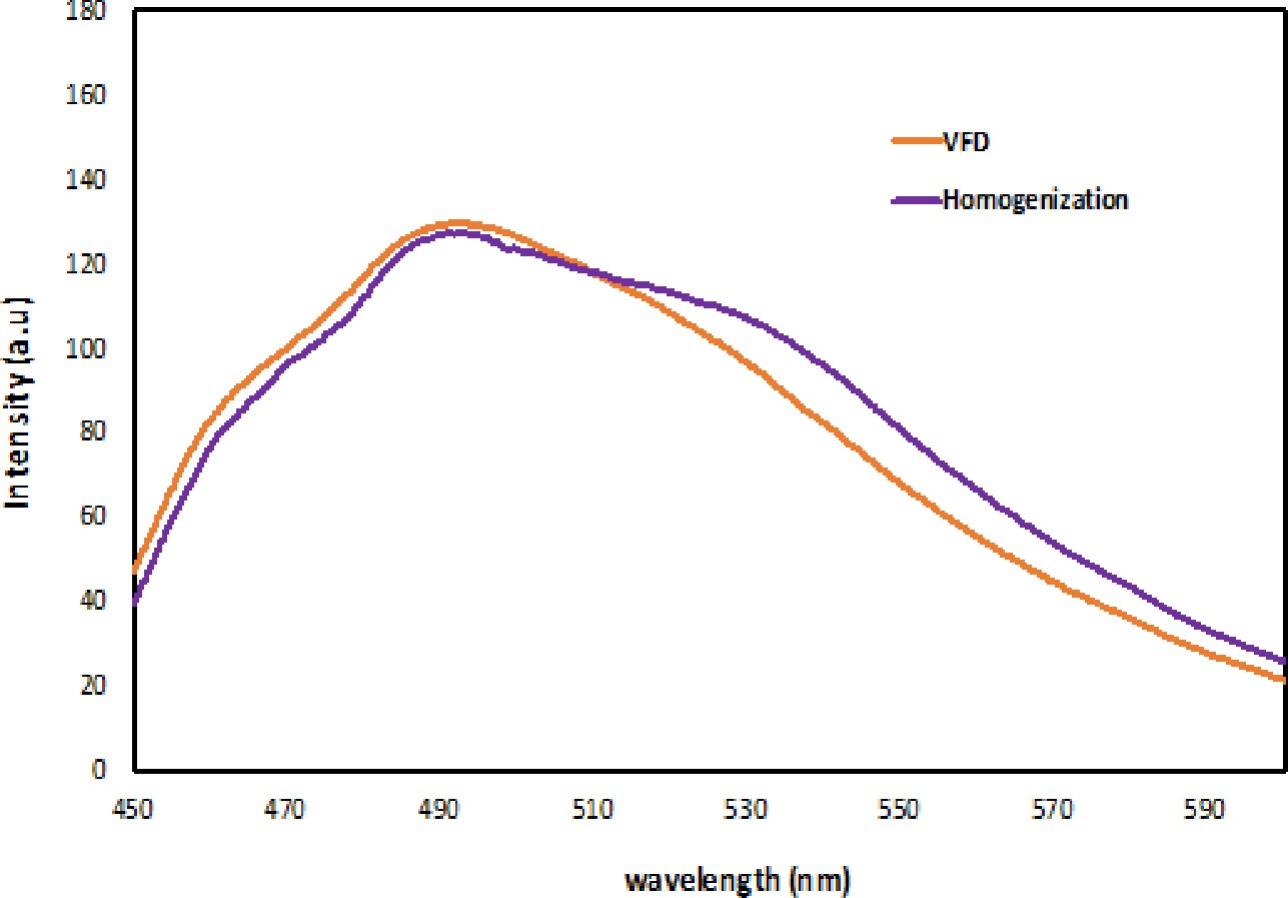
Fluorescent spectrums (path-length of quartz cuvette: 10mm; excitation band slits: 10 nm; emission band slits: 10 nm; scan rate: 1800 nm/min; excitation wavelength: 425 nm; emission spectra: 450–600 nm) for VFD and homogenization samples.

**Fig 7:**
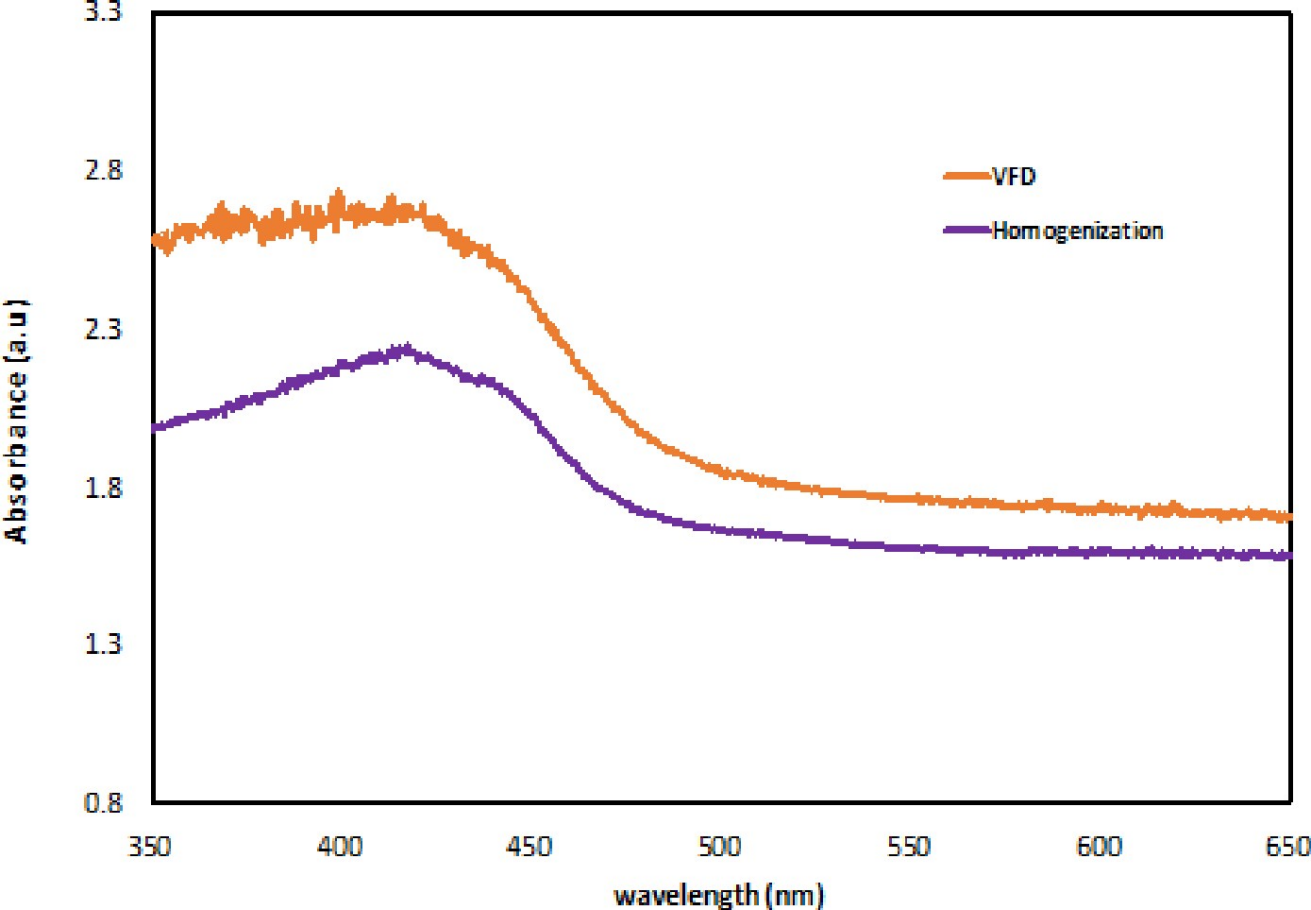
UV-visible spectroscopy (temperature: 25°C; path-length of quartz cuvette: 10 mm) for VFD and homogenization samples.

The effectiveness of encapsulation was further measured using fluorescent spectroscopy (Fig 6). Curcumin has intrinsic fluorescence with a wide intensity band between 540–550 nm in aqueous medium [20]. A shift is observed at 500 nm for both VFD and homogenization samples. This shift is indicative of interactions between sucrose mono-laurate and curcumin micelles. However, comparing with the VFD generated sample, the homogenization sample has a broader fluorescent spectrum curve at 530 to 540 nm. This is likely to be due to the presence of curcumin not encapsulated during the homogenization processing. UV-visible spectroscopy was also used to characterize the samples (Fig 7). Curcumin has a strong absorbance band at 425 nm associated with *π*–*π* excitation of the di-ketone rings [21]. A 5 nm blue shift is observed at 420 nm, highlighting a change in the molecular environment of the curcumin, noting curcumin in a more hydrophobic environment typically has such a blue-shift (Ming, *et*.*al*., 2015).

### 4.3 Pasteurization of raw milk

**Fig 8:**
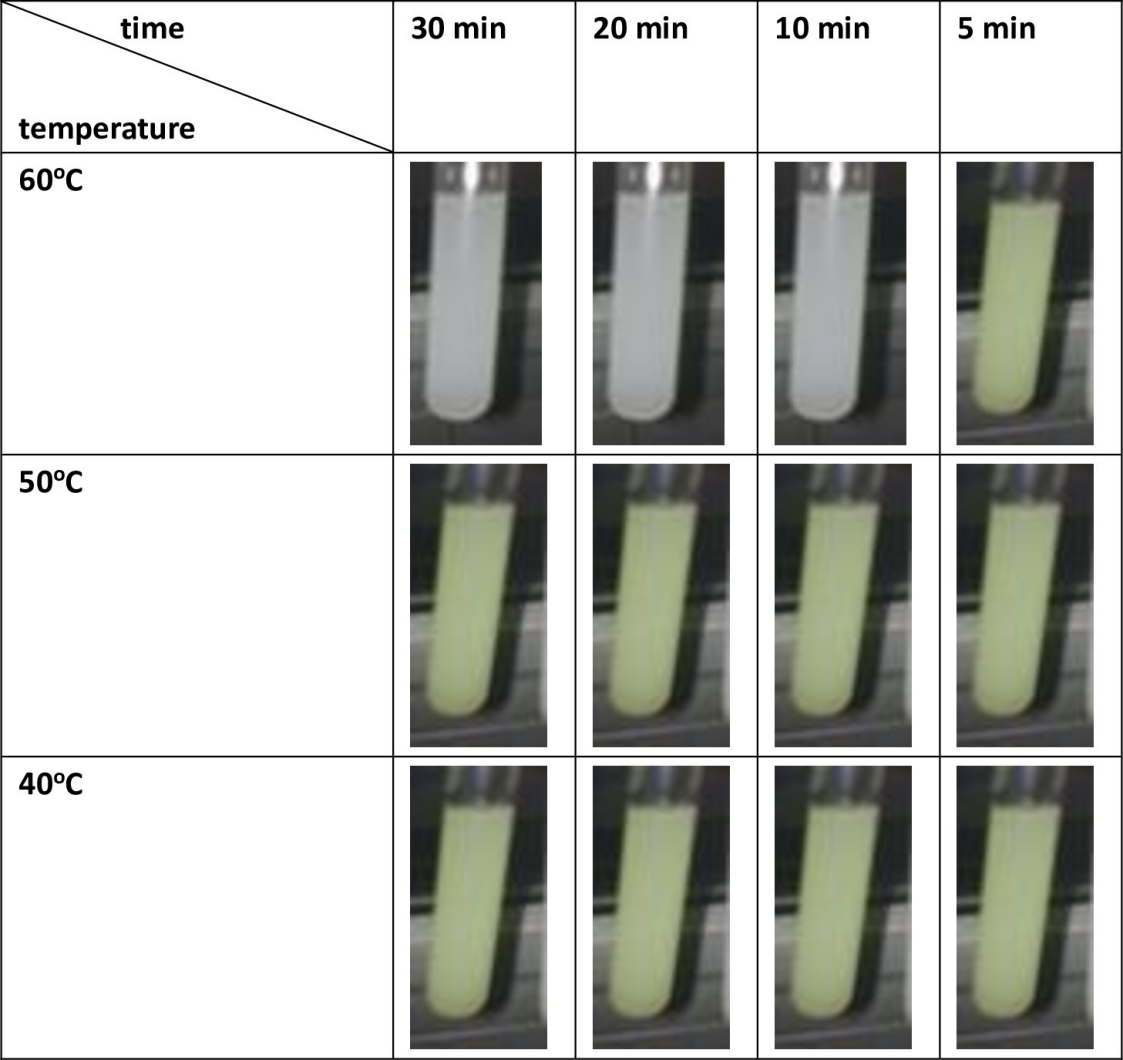
The effect of VFD-pasteurization with different processing times (5–30 min) and temperatures (40–60°C) on raw milk (VFD Mode: confined mode; VFD rotation speed: 9000 rpm; VFD tilt angle: 45°).

The effect of VFD-pasteurization with different processing times and temperatures on raw milk is shown in Fig 8. The white color in Fig 8 shows that there is no color change during the test, which would arise from complete deactivation of Alkaline Phosphatase. The lack of color change confirmed the complete pasteurization. In contrast, the yellow color in Fig 8 is indicative of incomplete pasteurization. The standard conventional pasteurization method requires the raw milk to be processed at 60°C for 30 minutes. As seen from Fig 8, the VFD-pasteurization is unable to reduce the pasteurization temperature, but it was effective in reducing the processing time from 30 minutes to 10 minutes. While this is still longer than the standard processing time for UHT, which is 1–2 seconds at 135°C, the shorter processing time in the VFD preserves the nutritional value. This aside, the UHT equipment costs approximately USD 1–2 million, considerable more than the cost of a VFD, as discussed above. Overall, VFD-pasteurization provides the balance between cost and nutritional value for milk pasteurization, but this will require addressing scaling up for the processing using, for example in using a larger diameter tube in the VFD.

## 5. Conclusion

We have established the utility of the VFD in food processing, for three different food models. The high heat transfer and shear stress (mechanoenergy) in the VFD is effective in shortening the processing time of enzymatic hydrolysis from about 2–3 hours to 20 minutes. In the same vein, the VFD was effective in reducing the processing time of standard pasteurization from 30 minutes to 10 minutes, and in reducing the size of encapsulated curcumin particles, from approximate 1000 nm to less than 100 nm, without requiring expensive processing homogenization equipment. This initial data and analysis demonstrates a potential of VFD processing in the food industry, noting that the device itself is relatively inexpensive.

## Supporting information

S1 Fig Photograph of the vortex fluidic device (VFD)-heating system highlighting its salient features. (DOCX)

S2 Fig Dynamic light scattering (DLS) data for preparing encapsulated particles using a vortex fluidic device (VFD) operating at different rotating speeds, for a fixed tilt angle and flow rate. (DOCX)

S3 Fig Dynamic light scattering (DLS) data for preparing encapsulated particles using a vortex fluidic device (VFD) operating at different flow rates, for a fixed tilt angle and rotation speed. (DOCX)

S4 Fig Dynamic light scattering (DLS) data for preparing encapsulated particles using a vortex fluidic device (VFD) operating at different tilt angles, with a fixed tilt angle and different flow rates. (DOCX)
