# Supplementary material for: Vortex fluidic mediated food processing
Source: PLoS One. 2019 May 30;14(5):e0216816. doi: 10.1371/journal.pone.0216816 (PMC6542520; doi:10.1371/journal.pone.0216816)
Supplement: S1 Fig — (DOCX) [file pone.0216816.s001.docx]

1. **Vortex Fluidic Device (VFD)-heating system applied for enzymatic hydrolyzation of protein in milk powder and pasterurization of raw milk.**


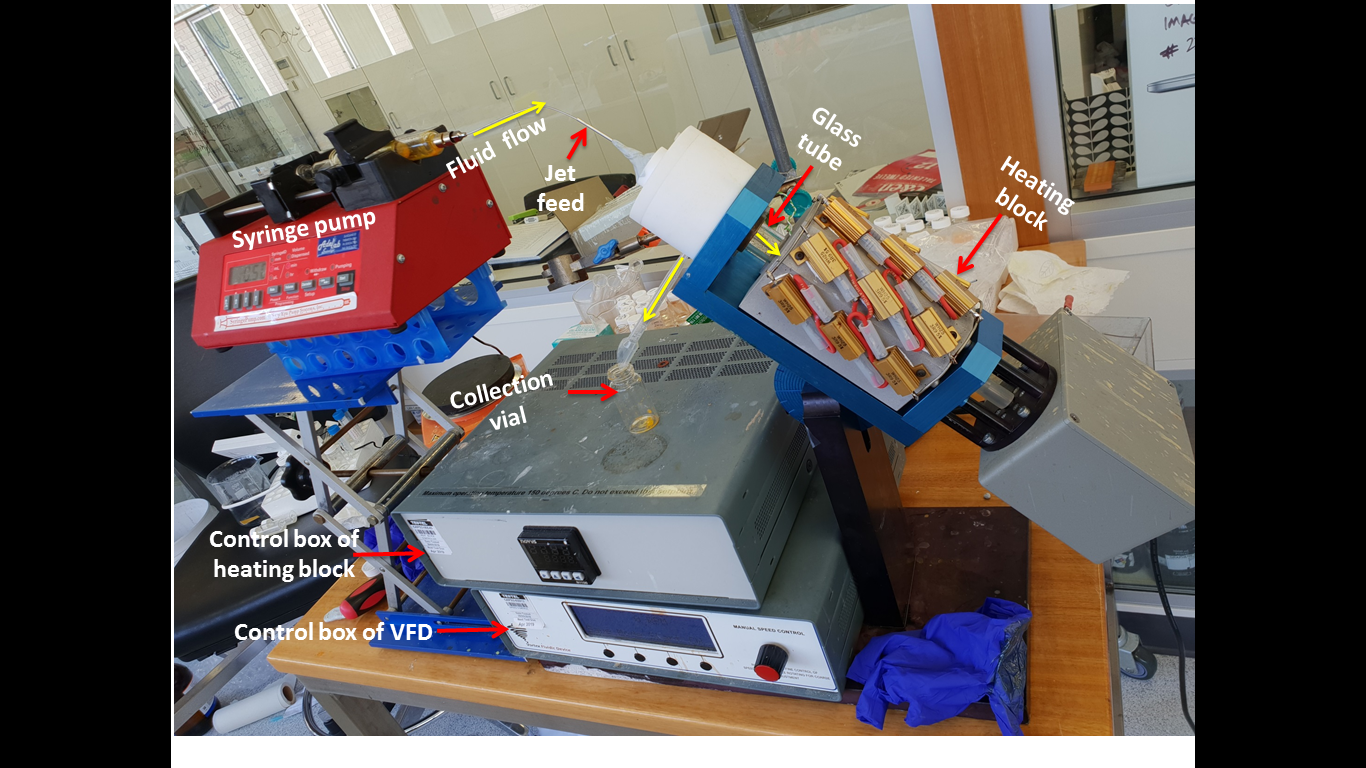


**S1 Fig.** Photograph of the vortex fluidic device (VFD)-heating system highlighting its salient features
