# Supplementary material for: Vortex fluidic mediated food processing
Source: PLoS One. 2019 May 30;14(5):e0216816. doi: 10.1371/journal.pone.0216816 (PMC6542520; doi:10.1371/journal.pone.0216816)
Supplement: S2 Fig — (DOCX) [file pone.0216816.s002.docx]

**Optimization of the rotational speed, flow-rate and tilt angle for curcumin encapsulation with fish oil and sucrose monolaurate as a emulsifier**

Nano-encapsulations were formulated using curcumin, as a bioactive ingredient, and a mixture of non-ionic surfactant and water. Briefly, 10 mg of curcumin was mixed and dissolved in 60 mg of fish oil. Then the curcumin-fish oil mixture was premixed with 10 mg of sucrose monolaurate and 10 mL of water by using a benchtop vortex mixer, and introduced into the borosilicate glass tube (20 mm OD) in the VFD through jet-feeds, with the tube rotating at 8000 rpm, at a flow-rate of 0.1 mL/min, with the tilt angle of the tube at 45^o^ and the device operating at room temperature. The solution was collected and sonicated for 20 minutes. The data leading up to this optimizing the rotating speed, flow-rate and tilt angle are shown below. The sample produced from optimized conditions was applied to the following UV-visible absorption spectroscopy measurements and fluorescence spectroscopy measurements.

**Speed optimization for curcumin encapsulation:**


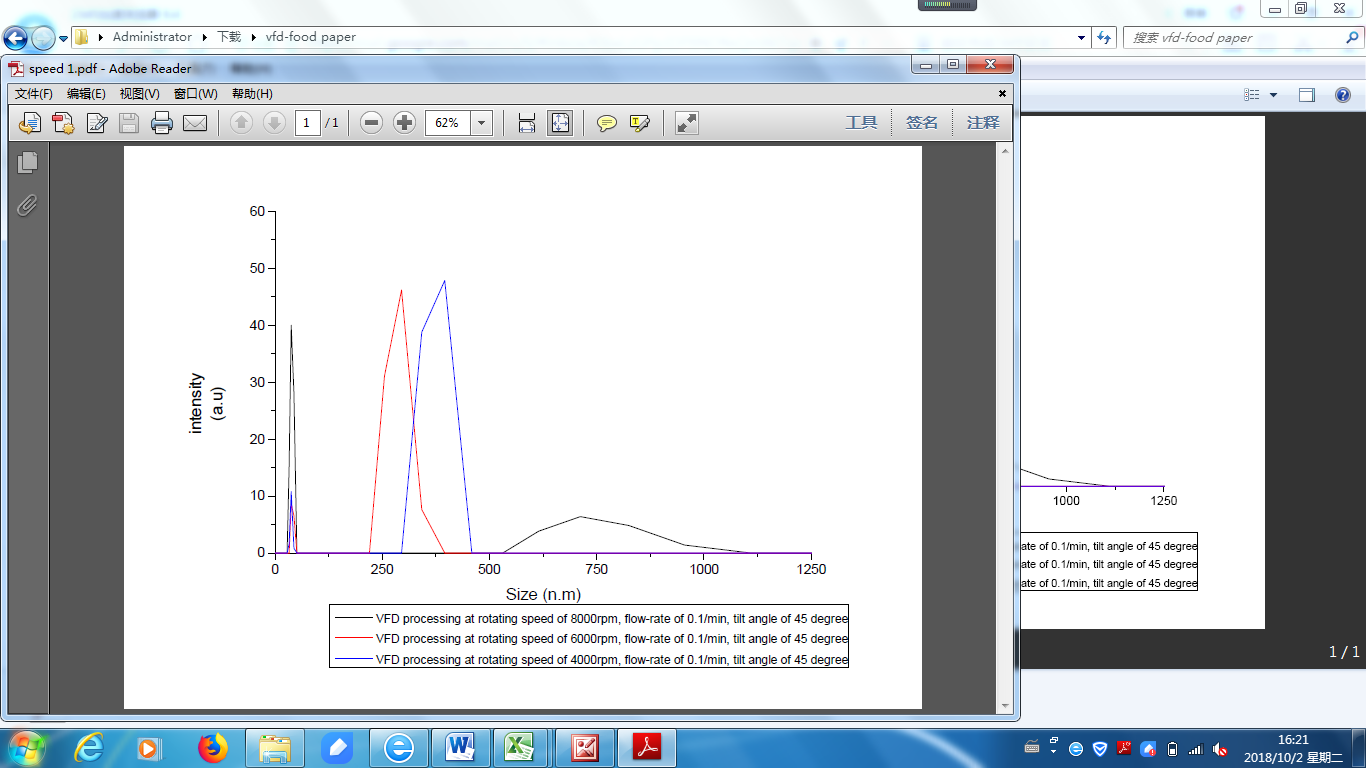


**S2 Fig.** Dynamic light scattering (DLS) data for preparing encapsulated particles using a vortex fluidic device (VFD) operating at different rotating speeds, for a fixed tilt angle and flow rate.
