# Supplementary material for: Vortex fluidic mediated food processing
Source: PLoS One. 2019 May 30;14(5):e0216816. doi: 10.1371/journal.pone.0216816 (PMC6542520; doi:10.1371/journal.pone.0216816)
Supplement: S3 Fig — (DOCX) [file pone.0216816.s003.docx]

**Flow-rate optimization for Curcumin encapsulation:**


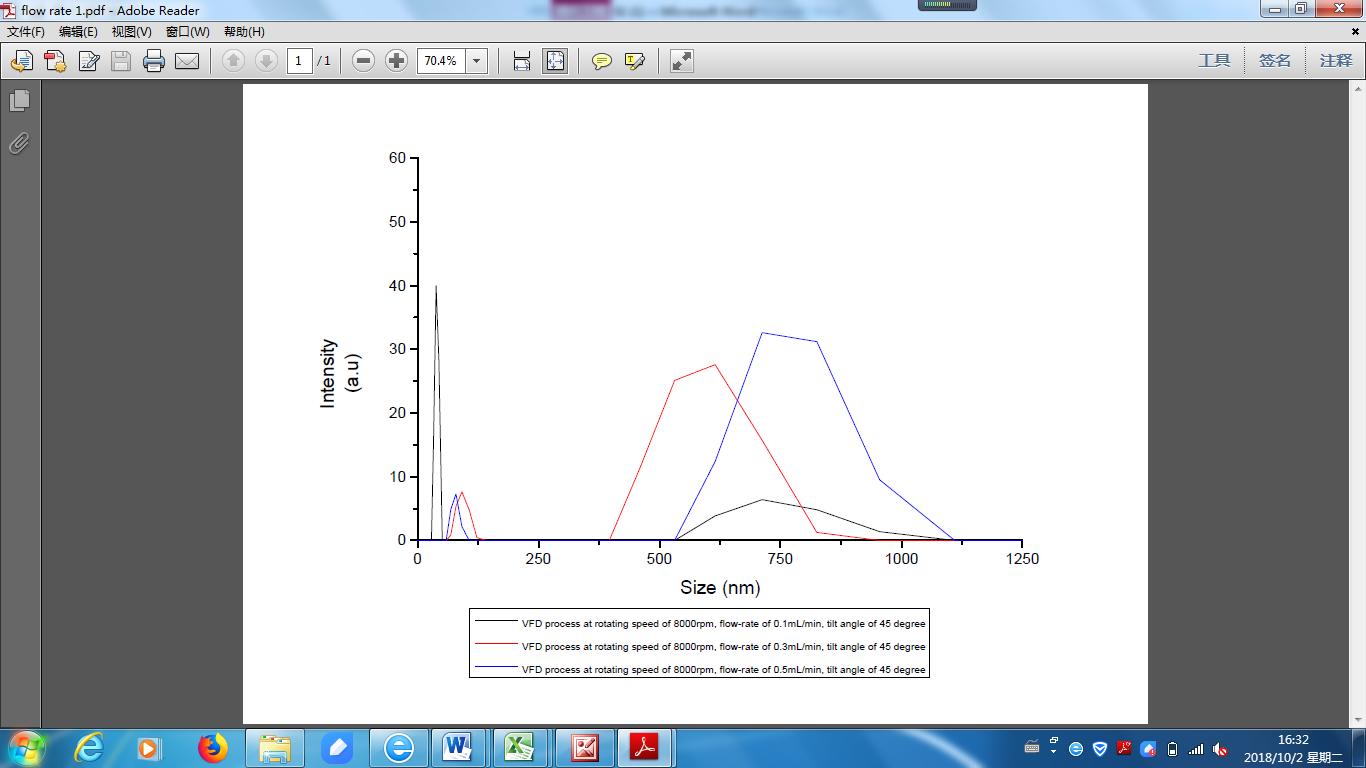


**S3 Fig.** Dynamic light scattering (DLS) data for preparing encapsulated particles using a vortex fluidic device (VFD) operating at different flow rates, for a fixed tilt angle and rotation speed.
