# Supplementary material for: Vortex fluidic mediated food processing
Source: PLoS One. 2019 May 30;14(5):e0216816. doi: 10.1371/journal.pone.0216816 (PMC6542520; doi:10.1371/journal.pone.0216816)
Supplement: S4 Fig — (DOCX) [file pone.0216816.s004.docx]

**Optimization of the tilt angle for curcumin encapsulation:**


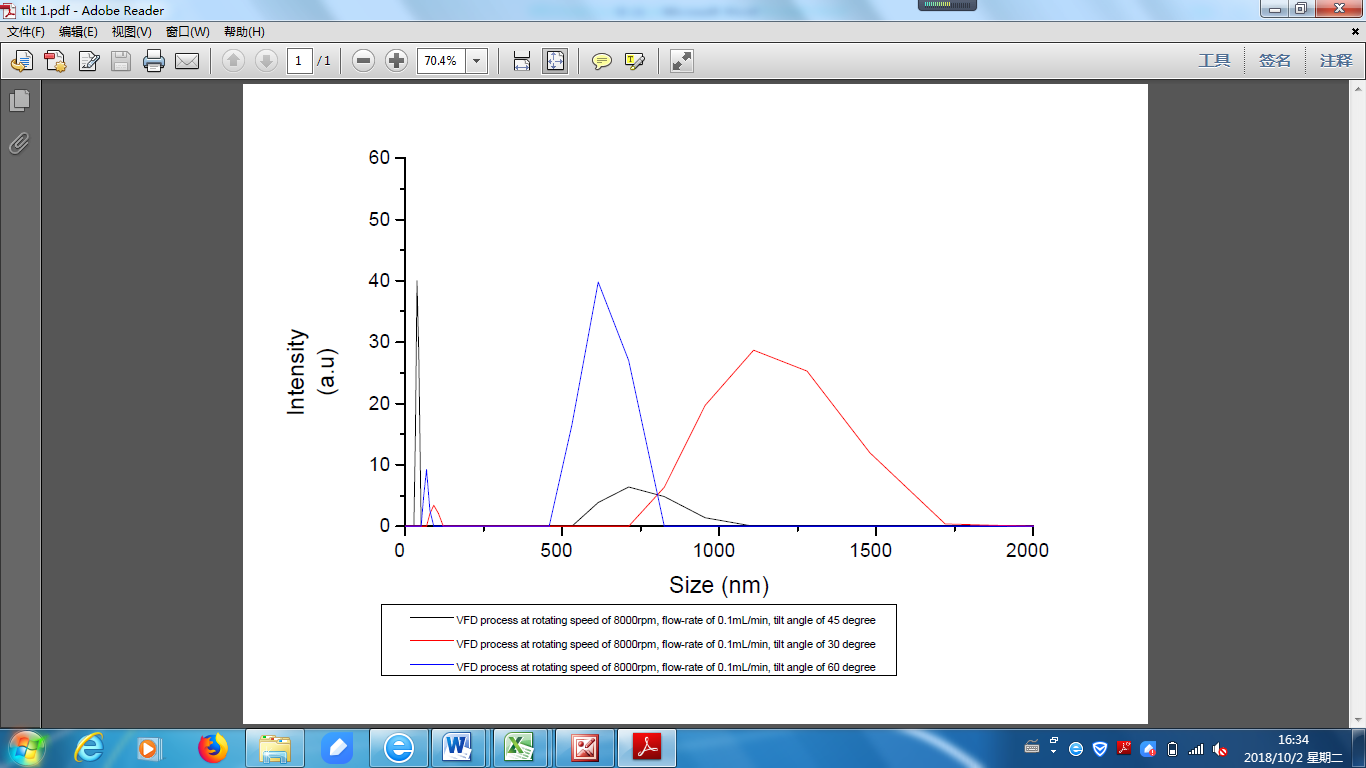


**S4 Fig.** Dynamic light scattering (DLS) data for preparing encapsulated particles using a vortex fluidic device (VFD) operating at different tilt angles, with a fixed tilt angle and different flow rates.
